# Comparison of Commensal *Escherichia coli* Isolates from Adults and Young Children in Lubuskie Province, Poland: Virulence Potential, Phylogeny and Antimicrobial Resistance

**DOI:** 10.3390/ijerph15040617

**Published:** 2018-03-28

**Authors:** Ewa Bok, Justyna Mazurek, Andrzej Myc, Michał Stosik, Magdalena Wojciech, Katarzyna Baldy-Chudzik

**Affiliations:** 1 Department of Microbiology and Genetics, Faculty of Biological Sciences, University of Zielona Góra, 65-561 Zielona Góra, Poland; j.mazurek@wnb.uz.zgora.pl (J.M.); andrzej.myc@gmail.com (A.M.); m.stosik@wnb.uz.zgora.pl (M.S.); k.baldy-chudzik@wnb.uz.zgora.pl (K.B.-C.); 2 Laboratory of Virology, Department of Immunology of Infectious Diseases, Ludwik Hirszfeld Institute of Immunology and Experimental Therapy, Polish Academy of Sciences, 53-114 Wroclaw, Poland; 3 Michigan Nanotechnology Institute for Medicine and Biological Sciences, University of Michigan Medical School, Ann Arbor, MI 48109-5648, USA; 4 Department of Mathematical Statistics and Econometrics, Faculty of Mathematics, Computer Science and Econometrics, University of Zielona Góra, 65-516 Zielona Góra, Poland; m.wojciech@wmie.uz.zgora.pl

**Keywords:** commensal *Escherichia coli*, adults, young children, virulence genes, phylogenetic grouping, antimicrobial resistance

## Abstract

Commensal *Escherichia coli* population is a dynamic structure which may be important in the pathogenesis of extraintestinal infections. The aim of this study was the comparison of genetic diversity of commensal *E. coli* isolates from two age group—adults and young children. *E. coli* strains were isolated on MacConkey agar and identified by biochemical tests. Determination of four major phylogenetic groups, identification of virulence genes and antimicrobial resistance determinants were performed by using multiplex or simplex PCR. Phenotypic analysis of resistance was based on disc-diffusion method. The prevalence of virulence genes was significantly higher among isolates from adults than from young children. Phylogroup B2 predominated among *E. coli* from adults, whereas phylogroup A was the most common in isolates from young children. The analyses of antimicrobial resistance revealed that resistance to at least one antimicrobial agent and multidrug-resistance were detected significantly more frequent in the isolates from adults than from young children. This study documented that the commensal *E. coli* isolates from adults showed greater genetic diversity than from young children and constitutes a substantial reservoir of the virulence genes typical for extraintestinal pathogenic *E. coli*.

## 1. Introduction

*Escherichia coli* bacteria is one of the most explored model organisms, partially due to its versatile nature. These bacteria constitute a component of the natural microbiota of warm-blooded animals including humans [1]. Some strains of the species are pathogens associated with intestinal or extraintestinal diseases in some cases leading to morbidity and mortality [2,3,4,5]. The vast diversity of this species results from the high recombination frequency and acquisition or loss of genetic information by horizontal gene transfer [1]. Such great plasticity of the genome speeds up the adaptation to new niches [6]. The origin of pathogenicity of *E. coli* remains unclear and it is unknown whether transition will go from commensal to pathogen or the other way around. Intestinal pathogenic *E. coli* strains (IPEC) are obligatory pathogens and can be easily distinguished from commensal or extraintestinal pathogenic *E. coli* (ExPEC) based on the presence of the selected virulence genes and phenotypic traits. ExPEC strains are facultative pathogens. They are part of healthy intestinal microflora, however they may become pathogenic in immune-deficient patients or during adaptation to other niches [3,7,8,9]. Various environmental factors may influence the emergence of virulence genes and antimicrobial resistance among commensal *E. coli* strains, and such strains constitute a reservoir of virulence and antimicrobial resistance [10,11,12].

*E. coli* strains colonize the infant’s intestine within a few hours after birth, likely deriving from the mother’s fecal microflora but also from maternity ward staff contact [13,14,15]. It was reported that each individual carries a single predominant strain. It is well known that the extraintestinal pathogenic strains that cause urinary tract infection, neonatal septicemia or meningitis belong mainly to phylogenetic group B2 and, to a lesser extent, group D [16,17]. Moreover, the strains of these phylogroups harbor more virulence factors than the strains of the A and B1 groups [17,18].

*E. coli* strains inhabiting the intestine of an individual can be divided into two groups: the resident strains that are present for months or years and the transient strains that appear and then disappear within a few weeks [19]. The resident *E. coli* strains, persisting in the intestinal microflora, more frequently carry the virulence genes encoding P fimbriae, K1 and K5 capsules, hemolysin, and other compounds responsible for iron acquisition such as aerobactin, compared with colonic transient strains in the same hosts [15,20]. These virulence genes are considered as encoding fitness factors to maintain *E. coli* in the human intestinal tract, not always associated with virulence [21].

The use of antibiotics in medicine and in the environment has caused intestinal microorganisms to become a reservoir of antibiotic resistance factors. Many recent studies revealed high, or increasing, incidence of antibiotic resistance in commensal *E. coli* from healthy children and adults coming from many countries [12,22,23]. Naturally, without the pressure of antibiotics in the gut, susceptible *E. coli* strains should have an advantage over the resistant ones. However it has been reported that the resistant *E. coli* are able to persist among intestinal microflora as well as susceptible strains [24].

Previous studies have reported the characteristics of commensal *E. coli* strains derived from adults [25,26], schoolgirls aged 7–16 [20,21] and from infants [15,24,27], whereas there is limited data in the literature regarding *E. coli* derived from young children aged 0.5–3. Moreover, it was also reported that the diversity and composition of microorganisms found in the intestine changes dramatically between birth and the age of 3 years [28]. It is important to ask if the pattern with dominance of phylogroup B2 in *E. coli* from infants, will still persists among young children. The age of 0.5–3 years is an interesting time period because the diet of young children is rather simple and consists of organic, less processed food as compared to adult diet. Besides, young children aged 0.5–3 are very active and interested in the surrounding world but not interested in hygiene practices, therefore they can acquire many *E. coli* strains from the environment. This is the first report on the comparison of genetic diversity of commensal *E. coli* isolates from two age group—adults and young children.

The aim of the present study was to characterize and compare commensal *E. coli* isolates derived from healthy adults and young children. The investigation encompassed genotypic identification of the virulence genes typically found in intestinal and extraintestinal pathogens, and the determination of the main phylogenetic structure of *E. coli*. Additionally, the prevalence of the antimicrobial resistance and resistance genes were determined. This study give the better insight into understanding of the relations between virulence genes, phylogenetic structure and the resistance genes of *E. coli* from adults and young children.

## 2. Materials and Methods

### 2.1. Sample Collection and E. coli Identification

A total of 382 fecal samples were collected from two groups of healthy humans living in Lubuskie Province (Western Poland). The first group consisted of 296 unrelated adult volunteers (from separate households) of both sexes (234 females and 62 males) aged 18–56 years (Figure S1), recruited mainly from students and university staff. The second group consisted of 86 unrelated young children (aged between 6 months and 3 years) of university staff and students attending the nursery. Freshly voided fecal samples were collected at home by the participants or by the participants’ parents, in sterile polystyrene containers. Samples from young children were collected in 2009–2010 and samples from adults were collected in 2012–2015. The fecal sampling procedure followed laboratory standards used in sampling for inoculation. The procedure ensured proper hygiene and posed no threat to volunteers. The volunteers themselves transported the samples to the laboratory. The samples were refrigerated upon arrival at the laboratory and processed the same day. After processing, samples were properly discarded. Every volunteer was adequately informed of the research aims. This study was approved by the bioethics committee at the District Medical Council in Zielona Góra (No. 03/85/2018). The volunteers completed a short questionnaire concerning: health, age, sex, diet and antibiotics usage within the period prior to sampling. The adults and young children used a regular diet appropriate to their age. The volunteers had not been treated with any antimicrobial agents within the three-month period prior to sampling. The stool samples were streaked on MacConkey agar and incubated at 37 °C for 24 h. The bacterial colonies showing a typical *E. coli* morphology were randomly selected and identified by biochemical IMVC tests (indole production, methyl red reaction, Voges-Proskauer test and citrate utilization). A single isolate representing each sample was randomly picked for further analysis. A single isolate was selected because it has been reported that individuals usually carry a predominant *E. coli* strain that constitutes more than half of the colonies isolated from stool sample [1,29]. It was also reported that the number of isolates sampled per individual (1, 5, or 10) did not affect the general pattern of phylogenetic distribution among the analysed populations [25]. Our pilot studies (data not shown) also confirm that most of the strains isolated from one stool samples were identical. All 382 *E. coli* isolates were stored frozen as a glycerol stock, at −80 °C, until the investigation. The DNA extraction was carried out using the thermal cell lysis method; 1.5–3 μL of the boiled bacterial supernatant was used as a template in all the PCR reactions.

### 2.2. Virulence Genotyping

The isolates were examined for the presence of 30 different intestinal and extraintestinal virulence associated genes, representing five functional categories presented in Table S1: List of virulence genes tested in this study. Multiplex PCR-based genotyping was performed with primers and conditions previously described [16,30,31,32,33,34,35,36]. The PCR amplification mixture in a volume of 25 μL contained: buffer solution (Thermo Scientific, Waltham, MA, USA); 2.5 mM MgCl_2_ (Promega, Madison, WI, USA); 0.5 mM of each dNTP (Promega); 0.2 μM of each primer (IDT, Coralville, IA, USA); 1 U of DyNAzyme II polymerase (Thermo Scientific) and 3 μL of DNA template. All PCR reactions included a negative control containing no DNA template and a positive control, containing DNA template from an *E. coli* isolates known to carry the identified gene (validated by sequencing). The PCR products were separated in 1.5% or 2% agarose gel electrophoresis and stained with ethidium bromide.

### 2.3. Phylogenetic Typing

The *E. coli* isolates were assigned to four major phylogenetic groups—A, B1, B2 or D based on the triplex PCR amplification method described by Clermont et al. [37].

### 2.4. Antimicrobial Susceptibility Testing

All the *E. coli* isolates were examined for their antimicrobial susceptibilities to 17 antimicrobial agents using the disc-diffusion method on Mueller Hinton agar (Merck, Darmstadt, Germany). The tests were performed following the European Committee on Antimicrobial Susceptibility Testing (EUCAST) standard methods [38]. The growth inhibition zone diameters were measured and interpreted according to the recommendations of the EUCAST [39] (for most antimicrobials) and the Clinical and Laboratory Standards Institute (CLSI) [40] (for cephalothin, cefotaxime, streptomycin, tetracycline, doxycycline, nalidixic acid and nitrofurantoin). The following antimicrobial agents were tested in this study: ampicillin (AMP, 10 µg), amoxicillin-clavulanic acid (AMC, 30 µg), piperacillin (PIP, 30 µg), cephalothin (CF, 30 µg), cefuroxime (CXM, 30 µg), cefotaxime (CTX, 30 µg), streptomycin (S, 10 µg), gentamicin (GM, 10 µg), amikacin (AN, 30 µg), tetracycline (TE, 30 µg), doxycycline (D, 30 µg), trimethoprim-sulfamethoxazole (SXT, 1.25/23.75 µg), chloramphenicol (C, 30 µg), nalidixic acid (NA, 30 µg), norfloxacin (NOR, 10 µg), ciprofloxacin (CIP, 5 µg), and nitrofurantoin (FM, 300 µg). The *E. coli* strains ATCC 25922 and ATCC 35218—a β-lactamase-producing strain were used as a negative and positive quality controls, respectively. The susceptible or intermediately susceptible isolates were considered to be susceptible. Multi-drug resistance (MDR) was defined as the non-susceptible profile to ≥1 agent in ≥3 antimicrobial categories. The antibiotic disks were purchased from Becton Dickinson (Franklin Lakes, NJ, USA).

### 2.5. Identification of Resistance Genes

The *E. coli* isolates that showed resistance to ampicillin, cefotaxime, streptomycin, tetracycline, trimethoprim/sulfamethoxazole and nalidixic acid were screened for the presence of genes encoding resistance to these antimicrobials. The following genes associated with resistance to beta lactams (*bla*_TEM_, *bla*_SHV_, *bla*_CTX-M_), streptomycin (*strA*/*strB*, *aadA1*), tetracycline (*tetA*, *tetB*, *tetC*), trimethoprim (*dfrA1*, *dfrA7*), sulfonamides (*sul1*, *sul2*, *sul3*), or quinolones (*qnrA*, *qnrB*, *qnrS*) were identified by PCR. The PCR reactions were carried out using previously published primers and conditions [41,42,43,44,45]. The PCR amplification mixture was prepared in a volume 25 μL as described above.

### 2.6. Statistical Methods

The presence of the virulence genes and antimicrobial resistance were categorized as 1 = present and 0 = absent. In order to determine whether there was a significant association between the prevalence of the virulence genes (as well as the antimicrobial agent) and the host (adults vs. young children) Pearson’s chi-squared test for independence or Fisher’s exact test were used. The Fisher’s exact test was used if more than 20% of the cells in the contingency table have expected frequencies below five. The evaluations of the frequency of the virulence genes among *E. coli* isolates from young children and adults within each phylogenetic group were tested using the chi-squared test for proportions or Fisher’s exact test for proportions, as appropriate. The null hypothesis assumes that the proportions in isolates from adults and young children are equal. The alternative hypothesis is one-sided and assumes that the proportion in one group (adults or young children) was lower or higher than in the other, as appropriate. For all the statistical tests the level of statistical significance was defined as 0.05.

The relations between the presence of a virulence gene and the age of individual persons (adults) were described using univariate logistic regression models. Given each model, the odds ratios (OR) and their 95% confidence intervals for the population were estimated. This relation is deemed statistically significant at the 0.05 level, if the confidence interval does not contain the value of 1.

In order to measure the strength of the associations for the cross tabulation of virulence genes and virulence genes with antimicrobial resistance of the *E. coli* isolates from both adults and young children, the Goodman and Kruskal tau coefficient was calculated.

Multiple correspondence analyses (MCA) were conducted to determine the overall relationship between the group of the host (adults, young children) and the characteristic of the isolates (the phylogenetic group and different profiles of the iron acquisition or protectins genes). The MCA were performed on the Burt table using the host’s age as the supplementary categorical variable. The statistical analyses were performed using the program R (R Core Team, R Foundation for Statistical Computing, Vienna, Austria).

## 3. Results

### 3.1. Frequency of Virulence Associated Genes

Two virulence determinants typical for IPEC pathotypes *escV* (EPEC, EHEC) and *east1* (EAEC), and 15 typical for ExPEC—*fimH*, *papA*, *sfaS*, *fyuA*, *iutA*, *iroN*, *ireA*, *kpsMT* II, *kpsMT* III, *ompT*, *traT*, *iss*, *cnf1*, *hlyA*, *agn43*—were identified among the analyzed isolates. The genes characteristic for the IPEC pathotypes—*bfpB* (EPEC), *ehxA* (EPEC, EHEC), *stx1*, *stx2* (EHEC), *eltA*, *estI*, *estII* (ETEC)—were not found in any of the tested isolates. Table 1 presents the distribution of the virulence genes in two groups of isolates, derived from adults and young children. Three of the detected virulence genes—*sfaS*, *escV*, *kpsMT* III—occurred only among the *E. coli* isolates from adults. The most common detected genes among tested functional category for the isolates from adults were: *fimH* (96.6%)—adhesins; *fyuA* (76%)—iron acquisition; *kpsMT* II (67.9%)—protectins, with the most frequent variant K2 (64.9%); and *cnf1* (21.6%)—toxins. In the biofilm formation category allele *K12* of the *agn43* gene showed the highest frequency of 59.5%. For the isolates from young children the following genes prevailed: *fimH* (88.4%)—adhesins; *fyuA* (41.9%)—iron acquisition; *traT* (43%)—protectins; and *east1* (25.6%)—toxins.

The most frequent variant in the group of *agn43* genes was K12, involving 61.6% of young children’s isolates. The comparison of the frequency of the isolates positive for particular virulence genes between the two tested groups showed statistically significant differences (*p* < 0.02) for most of the analyzed genes. The *E. coli* isolates derived from adults carried the virulence genes more frequently than isolates fr*om young children, with the exception of *east1* and agn43* K12 genes. There are no statistically significant differences between the two tested groups of *E. coli* for the *papA*, *sfaS*, *escV* and *agn43* K12 genes (Table 1).

### 3.2. Distribution of Virulence Genes among E. coli Isolates According to the Age of Adults

**Figure 1:**
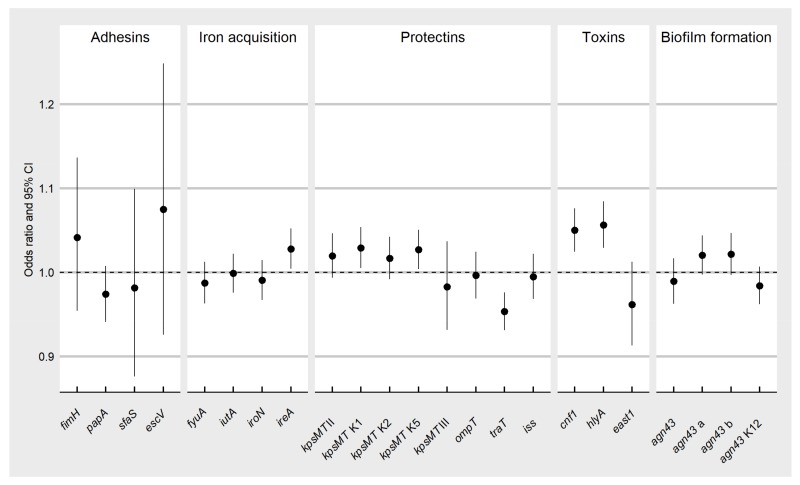
Odds ratios (the dots) and their 95% confidence intervals for the association of the occurrence of particular virulence genes in the *E. coli* isolates with the age of adults (18–56 years). The relations between the virulence gene and age of adults were described by logistic models. If the 95% CI is above the value of 1, this means that the ratio of the odds of the virulence gene’s occurrence increases with age; otherwise this ratio decreases with age. A narrow confidence interval indicates higher precision of the odds ratio.

An interesting question was whether there were any associations between the age of individuals and prevalence of virulence genes. The age range in the group of adults was high (from 18 to 56 years). Figure 1 presents the influence of adults’ age on the distribution of virulence genes in the *E. coli* isolates. The age of adults had a statistically significant effect on the prevalence of some genes (Figure 1). The chance of the presence of *ireA*, variants K1 and K5 of the *kpsMT* II, *cnf1* and *hlyA* genes increased with age and was 2.8% (95% CI: 1.004–1.052), 2.9% (95% CI: 1.005–1.054), 2.7% (95% CI: 1.004–1.05), 5% (95% CI: 1.024–1.076) and 5.6% (95% CI: 1.029–1.084) respectively, while for the *traT* gene the chance decreased with age and reached 5% (95% CI: 0.931–0.976). There was no statistically significant influence of the age of young children (the age ranged from 0.5 to 3 years) on the prevalence of virulence genes (data not shown).

### 3.3. Association between Virulence Genes

**Figure 2:**
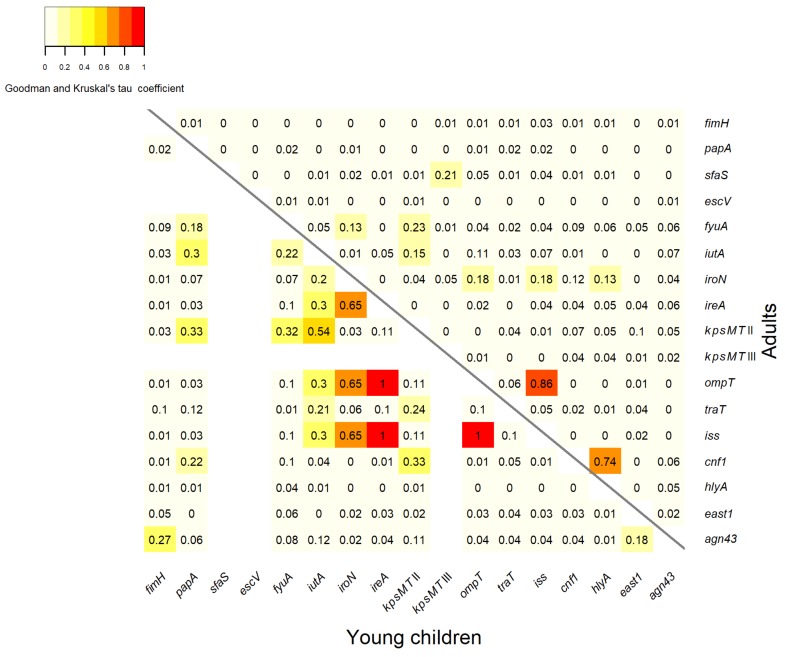
Statistical association between virulence genes of the *E. coli* isolates derived from adults (the part above the diagonal) and young children (the part under the diagonal). No values were introduced in the case of undetected genes.

The statistical analysis of the association between the virulence genes of the *E. coli* isolates is shown in Figure 2. A strong association occurred between the genes *cnf1* and *hlyA*, (association coefficient of 0.74), among the isolates from adults (Figure 2, the part above the diagonal). In the group of isolates from young children, very strong positive associations were found between the gene *ireA* and both genes *iss* and *ompT*, with association coefficients of 1. Strong associations were observed between the gene *iroN* and genes *iss*, *ompT*, *ireA*, with a coefficient of 0.65. The genes *iutA* and *kpsMT* II were also positively associated, with a coefficient of 0.54. There were moderate association between the gene *kpsMT* II and genes *cnf1*, *fyuA*, *papA*, with association coefficients of 0.33, 0.32 and 0.33, respectively, in the isolates from young children (Figure 2, the part under the diagonal). There is good evidence of a strong association between the genes *ompT* and *iss* among *E. coli* isolates from both adults and from young children, with coefficients of 0.86 and 1 respectively. Weak association, with coefficients ≤0.3, in the isolates from both young children and adults occurred between the remaining virulence genes.

### 3.4. Phylogenetic Structure of Commensal E. coli

**Figure 3:**
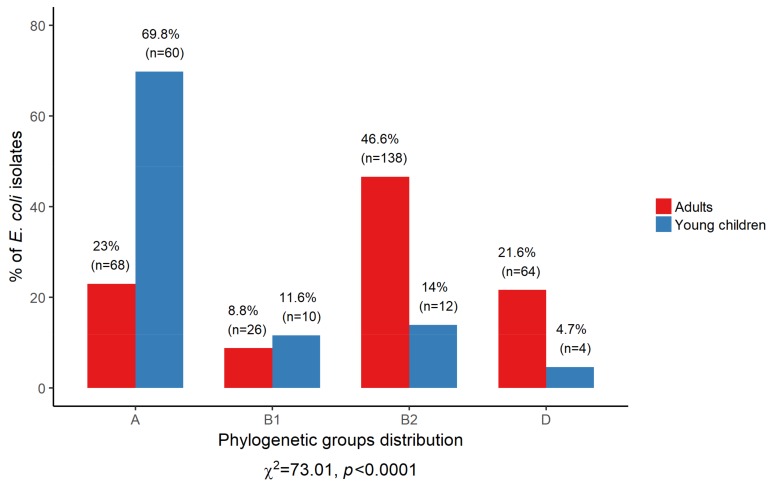
Phylogenetic structure of the *E. coli* isolates derived from two groups: adults and young children.

The *E. coli* isolates derived from adults and young children differed significantly in their assignments to the phylogenetic groups (*p* < 0.0001) (Figure 3). The phylogroups B2 and D significantly prevailed among the isolates from adults compared to young children, *p* < 0.0001 and *p* = 0.0005, respectively. Conversely, phylogenetic group A was significantly more frequent in the isolates from young children as compared to adults (*p* < 0.0001).

### 3.5. Distribution of Virulence Genes in Relation to Phylogenetic Groups

**Figure 4:**
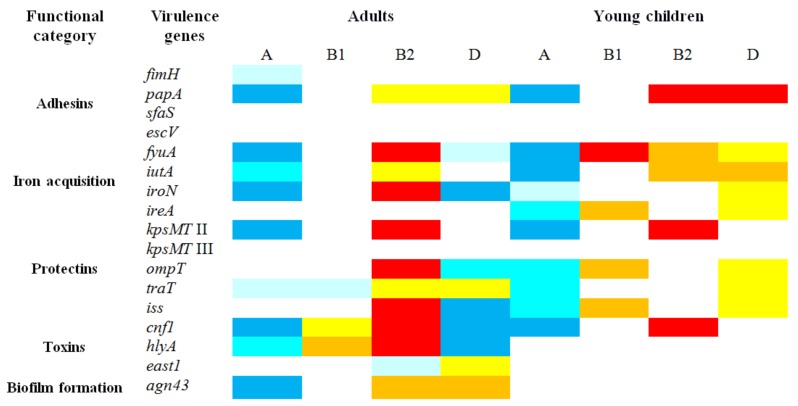
The frequency distribution of virulence genes among *E. coli* phylogroups was compared by Fisher’s exact test to its prevalence in a group consisted of the isolates of all the other phylogenetic groups. The values significantly higher than among the other groups are indicated as follows: 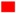 *p* < 0.001, 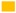 *p* < 0.01, 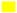 *p* < 0.05. The values significantly lower than among the other groups are indicated as follows: 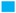 *p* < 0.001, 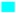 *p* < 0.01, 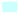 *p* < 0.05.

Figure 4 shows that the majority of the tested virulence genes were not randomly distributed among the four main phylogenetic groups of isolates. Generally, among the isolates derived from both adults and young children the frequencies of the particular genes were significantly lower (*p* < 0.05) in the isolates of group A, whereas the isolates of groups B2 harbored the virulence determinants significantly more frequently (*p* < 0.05).

The further analysis encompassed comparing the prevalence of different virulence genes between the isolates from adults and young children within each phylogenetic group. The results indicated that group A and B2 isolates and to a lesser extent group B1 from adults carried frequently virulence factors (*p* < 0.05) than the isolates from young children. The reverse situation was characteristic for the isolates of group D; several virulence genes were detected less frequently (*p* < 0.05) in the isolates from adults than from young children (Table 2). The *fyuA*, *iutA*, *ireA*, *kpsMT* II, *ompT*, *traT* and *iss* genes occurred more frequently in the isolates of group A from adults than from young children, while the *east1* gene was more frequent detected among the isolates from young children. The three genes *cnf1*, *hlyA* and *agn43* occurred more frequently in the isolates of group B1 from adults. The genes for *iroN*, *ireA*, *ompT*, *iss* and *hlyA* were found more frequently among the isolates of group B2 from adults than from young children; the one exception was the *papA* gene detected more often in the *E. coli* from young children. The *papA*, *iroN*, *ompT* and *iss* genes occurred less frequently in the isolates of group D from adults than from young children (Table 2).

### 3.6. Multiple Correspondence Analysis

**Figure 5:**
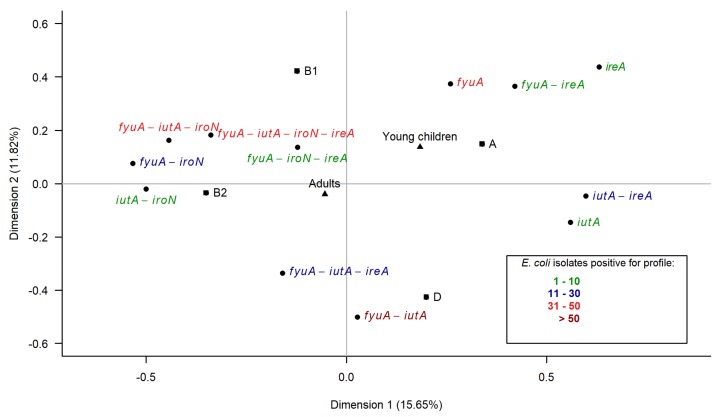
Multiple correspondence analysis (MCA) of the characteristics of the isolates (the phylogenetic group and different profiles of the iron acquisition genes) regarding two groups of the host (adults, young children) among 382 *E. coli* isolates from humans. The projection of the variables on the dimension1–dimension2 plane: the age of the host (adults, young children) is denoted by triangles, the phylogenetic group (A, B1, B2, D) by squares, the profiles (combinations of the iron acquisition genes *fyuA*, *iutA*, *iroN*, *ireA*) by circles. The number of isolates positive for a particular profile is distinguished by color.

The virulence genes within two categories—iron acquisition and protectins—were particularly prevalent among the tested *E. coli* isolates. To determine the overall relationships between two groups of the host (adults, young children) and the characteristics of the isolates, multiple correspondence analysis (MCA) was performed. The first analysis concerned the iron acquisition category. The projection of the variables on the dimension1–dimension2 plane accounted for 27.47% of the total variance and distinguished two major associations (Figure 5). The first association was positioned on the negative values of the first dimension and the positive and negative values of the second dimension and encompassed the isolates from adults, phylogenetic groups B1 and B2 variables and five complex iron acquisition profiles, consisting of two, three or four genes. The isolates from young children, the phylogenetic group A variables and three simple iron acquisition profiles consisting of one or two genes formed the second association and were located on the positive values of both dimensions. This analysis clearly distinguished adults, phylogroups B1 and B2 and the complex iron acquisition profile variables on the negative values of the first dimension from the young children, phylogroups A and D and the simple iron acquisition profile variables on the positive values of the first dimension.

**Figure 6:**
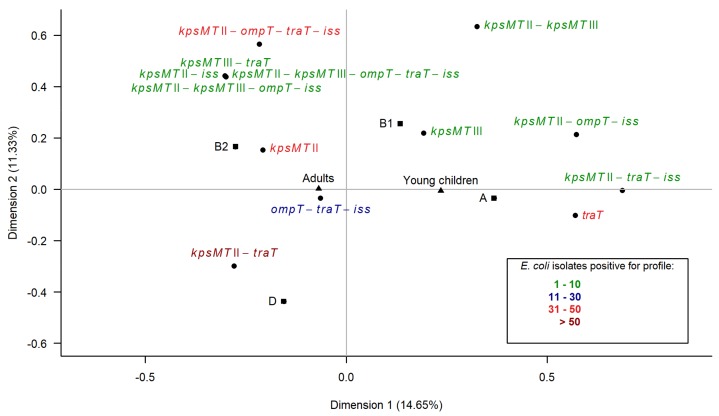
Multiple correspondence analysis (MCA) of the characteristics of the isolates (the phylogenetic group and different profiles of the protectin genes) regarding two groups of the host (adults, young children) among 382 *E. coli* isolates from humans. The projection of the variables on the dimension1–dimension2 plane: the age of the host (adults, young children) is denoted by triangles, the phylogenetic group (A, B1, B2, D) by squares, the profiles (combinations of the protectin genes: *kpsMT* II *kpsMT* III, *ompT*, *traT*, *iss*) by circles. The number of isolates positive for a particular profile is distinguished by color.

The second analysis concerned the protectin category. The projection of the variables on the dimension1–dimension2 plane accounted for 25.98% of the total variance (Figure 6). The first association encompassed the isolates from adults, phylogroup B2 and D variables and eight protectin profiles consisting of one to five genes, located on the negative values of the first dimension. The second association was formed by the isolates from young children, phylogroup A and B1 variables and five profiles consisting of one to three protectin genes, positioned on the positive values of the first dimension. The most complex protectin profiles were located close to phylogroup B2 and the isolates from adults’ variables. The analysis clearly distinguished adults, phylogroups B2 and D and the complex protectin profile variables from the young children, phylogroups A and B1 and the simple protectin profile variables.

### 3.7. Prevalence of Antimicrobial Resistance

Resistance rates to 17 antimicrobial agents were compared between the *E. coli* isolates from adults and young children and presented in Table 3. The most frequent resistance was detected for ampicillin (38.2% vs. 31.4%), cephalothin (29.1% vs. 27.9%), streptomycin (46.3% vs. 22.1%) and tetracycline (21.3% vs. 19.8%) in the isolates from adults and young children respectively. The resistance rates for norfloxacin and ciprofloxacin (2.7% vs. 2.3%) and cefotaxime (1.4% vs. 2.7%) were the lowest among the isolates from adults and young children respectively. Significantly higher frequency (*p* < 0.01) in resistance to amoxicillin/clavulanic acid, piperacillin and streptomycin were observed in isolates from adults as compared with young children. Moreover, resistance to at least one antimicrobial agent (75.7% vs. 54.7%) and multidrug-resistance (30.4% vs. 14%) were detected significantly more frequent (*p* = 0.0002 and *p* = 0.0024 respectively) in the isolates from adults than from young children (Table 3).

### 3.8. Detection of Resistance Determinants

The isolates were screened according to their resistance phenotypes for the carriage of a set of commonly encountered resistance genes. Genetic determinants associated with resistance to ampicillin, cefotaxime, streptomycin, tetracycline, sulfamethoxazole, trimethoprim and nalidixic acid were tested in this study. There were no statistically significant differences between the frequencies of the resistance determinants among the resistant *E. coli* isolates from adults and young children (Table S2). The most frequently observed gene encoding resistance to ampicillin was *bla*_TEM_ with 47.8% vs. 48.1% for adults’ and young children’s isolates, respectively. The gene encoding the extended spectrum β-lactamase *bla*_CTX-M_ was detected in all the isolates resistant to cefotaxime. Regarding streptomycin resistant *E. coli* the *aadA1* gene occurred most often (8.8% vs. 21.1%, in the isolates from adults and young children, respectively). The predominant gene among the tetracycline resistant isolates was *tetB* with the frequencies 50.8% vs. 41.2% for adults’ and young children’s isolates, respectively. The genes *dfrA7* (54.5% vs. 33.3%) and *sul2* (60.6% vs. 25%) prevailed among the trimethoprim/sulfamethoxazole resistant isolates from adults and young children, respectively. The *sul3* gene was not detected in any isolates. The *qnrS* gene was predominant among the *E. coli* resistant to nalidixic acid (17.9% vs. 25%, in the isolates from adults and young children, respectively). The *qnrA* gene was not detected in any isolates.

### 3.9. Correlations between Virulence Genes and Antimicrobial Resistance

The results revealed that the associations between the virulence genes and antimicrobial resistance were weak (association coefficients < 0.2) for *E. coli* isolates from adults and young children.

## 4. Discussion

To the best of our knowledge, this is the first study to compare the genetic diversity of commensal *E. coli* isolates collected from two different age groups (adults and young children). *E. coli* isolates from fecal samples were characterized in terms of the presence of virulence genes, phylogenetic structure, and antimicrobial resistance. Our results indicate that *E. coli* isolated from adults differ in their phylogenetic structure, harbour a greater variety of virulence genes, and multidrug-resistance phenotypes, compared to *E. coli* from young children.

Our results have shown that commensal *E. coli* isolates from healthy humans constitute a substantial reservoir of genes related to the extraintestinal pathotypes. All of the 15 tested virulence genes typical for ExPEC were detected and, what is important, the prevalence of these genes was significantly higher among the isolates from adults than from young children. In contrast, only two of the nine tested virulence genes associated with the intestinal pathotypes were found: *escV* and *east1*. The *escV* gene was identified only in one isolate from adults, while the *east1* gene was more common and was detected more frequent among the *E. coli* isolates from young children than adults. The previous studies also reported that the presence of the intestinal virulence markers actually was associated with pathogenesis [2,3,46,47]. The extraintestinal virulence genes such as those encoding the fimbrial adhesins, iron acquisition systems, protectins or toxins occur not only in pathogens, but also in commensal microflora of healthy people [3,9,21,47,48]. Previous reports indicated that virulence genes associated with extraintestinal pathogenesis in fact help the *E. coli* strains to colonize the human gut; therefore they may be considered as a fitness factor and the virulence is a coincidental side effect [8,21,48].

The question arises whether the adult’s age has also an influence on the prevalence of virulence genes. The analysis revealed that there are no statistically significant differences in the distribution of virulence genes in the *E. coli* isolates in relation to the age of the adult host. The only exception was some genes from the categories of iron acquisition (*ireA*), protectins (variants K1 and K5 of the *kpsMT* II) and toxins (*cnf1*, *hlyA*), which tend to accumulate in *E. coli* over the life time of the host. The opposite situation occurred only for the *traT* gene; the chance of accumulation decreases with age. Overall, the results suggest that the difference in prevalence of virulence genes is not correlated with age of adults, however, there is difference in prevalence of virulence genes between adults and young children.

The phylogenetic analysis revealed that the distribution of four main *E. coli* phylogroups differ significantly (*p* < 0.0001) between the isolates derived from adults and from young children. *E. coli* assigned to phylogroup B2 dominated among adults, whereas phylogenetic group A isolates prevailed among young children. The distribution of the phylogroups among the human population represents two patterns, the first with predominance of group A typical for European population (in 1980s), Africa, South America and Asia [1,25,26,49], and the second pattern that the isolates of group B2 prevailed is characteristic for Europe in the 2000s, North America, Japan and Australia [1,25]. The changes in the phylogenetic structure of the *E. coli* isolates from Europe during the period of 20 years may be the result of the increasing of food processing and change in dietary habits [1,25]. The results of the present study with the dominance of phylogroup B2 among *E. coli* isolates from adults are consistent with the previous reports presenting the second pattern of phylogenetic structure, whereas the predominance of isolates of phylogroup A among young children is in accordance with the first pattern [1,25,26,49]. The diet of young children aged 0.5–3 years is rather simple and in the most part consists of organic, less processed food as compared to adult diet. The host diet may be a key factor which had an influence on the phylogenetic structure of the *E. coli* isolates.

The analysis of the distribution of the virulence genes in relation to the phylogenetic groups revealed that the *E. coli* group B2 isolates were an extensive reservoir of virulence genes while group A was not.

Our results suggest that the higher frequencies of the virulence genes in the *E. coli* isolates from adults is due to predomination of phylogroup B2, therefore the comparison between the *E. coli* isolates from adults and young children with respect to the frequency of the virulence genes in each phylogroup was performed. The results showed that the *E. coli* isolates from adults assigned to either phylogroup A or B2 carried statistically significant (*p* < 0.05) more virulence determinants, particularly associated with iron acquisition and protection, than the isolates from young children assigned to phylogroup A and B2. Three genes from the toxin and biofilm formation categories occurred more frequently among phylogroup B1 isolates from adults than from young children. Our results documented that the genetic diversity of the isolates of phylogroups A, B2 and to a lesser extent B1 differs between adults and young children. The isolates may acquire genetic diversity through horizontal gene transfer. Possibly the resident strains that persist longer time in the intestinal tract have a greater chance to undergo numerous horizontal transfer events resulting in accumulation of the virulence determinants. Our studies may suggest that the strains of phylogroup D from adults are less diverse regarding the virulent determinants than *E. coli* strains from young children, but these results should be confirmed on a larger number of subjects.

Virulence genes are often clustered in the chromosomal genomic regions acquired by horizontal gene transfer called pathogenicity islands [10,50], or in large plasmidic regions [51,52]. One such region typical for the ExPEC plasmids has been recently described and called the conserved virulence plasmidic (CVP) region. This region harbors eight virulence genes, including *iroN*, *ompT* and *iss* [52,53]. The results regarding the associations between the virulence genes revealed that there were fewer statistically significant associations among the *E. coli* from adults than from young children. Strong associations were found between the *iroN*, *ompT* and *iss* genes among the isolates from young children. In the *E. coli* isolates from adults there remained only associations between the *ompT* and *iss* genes. The prevalence of these genes was much higher among the *E. coli* isolates from adults than among the isolates from young children. These results suggested that there was a tendency for accumulation of these genes in different combinations in the isolates from adults. This observation indicated that in commensal *E. coli* strains from adults there is low probability of presence of a specific pathogenic plasmidic CVP region. Two genes from the toxin category—*cnf1* and *hlyA*—were strongly associated among the isolates from adults. What it was previously reported, these genes may be concealed together on pathogenicity island PAI II_J96_ in pathogenic strains [10]. The existence of PAI II_J96_ in our set of *E. coli* isolates from adults will be confirmed in near future.

Previous studies revealed that the ability of some *E. coli* strains to persist in the colonic microbiota may be related to the carriage of the virulence genes for P fimbriae, aerobactin (*iutA*), yersiniabactin (*fyuA*) and capsular antigen (*kpsMT* II variants K1 and K5) and that these genes may play a key role as fitness factors [15,20,21,48]. In our studies the results of MCA analyses showed that the isolates from adults correspond primarily to phylogroup B2 and these variables were positioned close to the isolates positive for the more complex and also more numerous profiles both the iron acquisition and protectin categories. The *E. coli* isolates from young children correspond strongly to phylogroup A and to the isolates with simpler profiles. These results confirmed that the *E. coli* isolates derived from adults exhibited more genetic diversity than isolates from young children. There were isolates carrying three, four and even five genes of the same functional category. This indicated that the *E. coli* isolates derived from adults, particularly of phylogroup B2, constitute a significant reservoir of extraintestinal virulence genes and have a tendency to collect them.

The comparison of the resistance rates to 17 antimicrobial agents revealed that the *E. coli* isolates showed significantly higher (*p* < 0.01) resistance to three antibiotics: amoxicillin/clavulanic acid, piperacillin and streptomycin in adults as compared to young children. Likewise, significantly more isolates from the adults than from the young children were classified as strains resistant to at least one antimicrobial agent (*p* = 0.0002) and to multidrug-resistant strains (*p* = 0.0024). These results suggested that the older the host, the higher accumulation of resistance determinants in *E. coli* strains occurs but to a lesser degree as in the case of the virulence genes.

Our results indicated that the prevalence of resistance to ampicillin, cephalothin, streptomycin and tetracycline among the *E. coli* isolates from both adults and young children was rather moderate in the region of Western Poland, prevalence of resistance to trimethoprim/sulfamethoxazole was low/moderate, whereas the rates of resistance to the third-generation cephalosporin cefotaxime and fluoroquinolones were low. Depending on the country and different regions of the world, higher [22,23,49,54,55,56], similar [23,54,55] or lower [24,49,54,57] rates of resistance to the antibiotics mentioned above were observed among *E. coli* isolates from adults and children. The fact that the *E. coli* isolates from young children exhibited moderate prevalence of resistance to tetracycline, a drug which should not be administered to children, may be a consequence of the previous exposure of food animals to growth-promoting antibiotics and the dissemination of these agents in the environment [58,59,60]. Some previous studies have revealed that resistant bacteria can be found even in conventional retail foods and ready-to-consume items, which suggests that humans are often exposed to such bacteria through daily food intake [61,62,63].

All the resistant *E. coli* isolates were screened for the carriage of a set of commonly encountered resistance determinants. The results revealed no statistically significant differences between the frequencies of the tested resistance genes in the resistant *E. coli* isolates from adults and young children. It was probably due to the similar rates of resistance to most of the tested antibiotics in these two groups of isolates. The *bla*_TEM_, *aadA1*, *tetB*, *dfrA7*, *sul2* and *qnrS* genes related to ampicillin, streptomycin, tetracycline, trimethoprim/sulfamethoxazole and quinolone resistance respectively were the most common. The resistant isolates negative for the tested resistance determinants may have carried one of the other resistance genes.

Previous studies have reported that strains carrying the *papC* or *iutA* gene were more often resistant to at least one of the tested antibiotics than were strains without these traits [24,64]. Virulence genes and resistance genes may occur together on mobile genetic elements and be co-selected. The results of these studies revealed that the association between the virulence genes and antimicrobial resistance were weak in both groups of *E. coli* isolates.

## 5. Conclusions

This study analyzed the relationships between the distribution of virulence genes, phylogenetic structure and prevalence of antimicrobial resistance among *E. coli* isolated from adults and young children in Lubuskie province, Poland. This is the first such comparison regarding only commensal strains. The results revealed significant difference in the distribution of the virulence genes, phylogenetic groups as well as the multidrug-resistance among *E. coli* from adults and young children. This study confirmed that the commensal *E. coli* isolates, particularly that derived from adults, constitutes a considerable reservoir of the virulence genes typical for ExPEC. The comparison of the frequency of the virulence genes between the *E. coli* isolates from adults and young children in relation to each phylogroup showed that greater genetic diversity exists in isolates from adults. This may suggest accumulation of the virulence determinants among commensal *E. coli* strains through horizontal gene transfer over the years. Further research will be needed to better understand the relationships between environmental factors and the genetic diversity of *E. coli* commensal strains. Plasticity of *E. coli* genome provides the ability to adapt to different nutrient conditions and niches in the host’s body [1,6]. An interesting question remains how the environmental conditions (diet, travel, cultural background, etc.) effect the shift of *E. coli* from commensal to pathogen strain responsible for extraintestinal infection.

## Figures and Tables

**Table 1:** Prevalence of virulence genes by functional categories among *E. coli* isolates from healthy adults and young children.

Functional Category Virulence Gene	*E. coli* Pathotype	Number (%) of *E. coli* Isolates with Virulence Genes		Test of Independence *p*-Value
		Adults	Young Children	
		*n* = 296	*n* = 86	
**Adhesins**				
*fimH*	ExPEC	286 (96.6)	76 (88.4)	0.005 *
*papA*	ExPEC	54 (18.2)	10 (11.6)	0.1482
*sfaS*	ExPEC	4 (1.4)	0	0.5787
*escV*	EPEC, EHEC	1 (0.3)	0	1
*bfpB*	EPEC	0	0	-
**Iron acquisition**				
*fyuA*	ExPEC	225 (76)	36 (41.9)	<0.0001 *
*iutA*	ExPEC	184 (62.2)	17 (19.8)	<0.0001 *
*iroN*	ExPEC	110 (37.2)	4 (4.7)	<0.0001 *
*ireA*	ExPEC	87 (29.4)	6 (7)	<0.0001 *
**Protectins**				
*kpsMT* II	ExPEC	201 (67.9)	16 (18.6)	<0.0001 *
-K1	ExPEC	158 (53.4)	10 (11.6)	<0.0001 *
-K2	ExPEC	192 (64.9)	15 (17.4)	<0.0001 *
-K5	ExPEC	125 (42.2)	10 (11.6)	<0.0001 *
*kpsMT* III	ExPEC	18 (6.1)	0	0.02 *
*ompT*	ExPEC	62 (21)	6 (7)	0.003 *
*traT*	ExPEC	190 (64.2)	37 (43)	0.0004 *
*iss*	ExPEC	70 (23.6)	6 (7)	0.0007 *
**Toxins**				
*cnf1*	ExPEC, NTEC	64 (21.6)	6 (7)	0.002 *
*hlyA*	ExPEC	50 (16.2)	4 (2.3)	0.004 *
*east1*	EAEC	28 (9.5)	23 (25.6)	<0.0001 *
*ehxA*	EPEC, EHEC	0	0	-
*stx1*	EHEC	0	0	-
*stx2*	EHEC	0	0	-
*eltA*	ETEC	0	0	-
*estI*	ETEC	0	0	-
*estII*	ETEC	0	0	-
**Biofilm formation**				
*agn43*	ExPEC	240 (81.1)	58 (67.4)	0.007 *
-*a*	ExPEC	106 (35.8)	12 (14)	0.0001 *
-*b*	ExPEC	73 (24.7)	4 (4.7)	<0.0001 *
-*K12*	ExPEC	176 (59.5)	53 (61.6)	0.7179

* Statistically significant.

**Table 2:** Comparison of the frequency of virulence genes among the *E. coli* isolates from adults and young children within each phylogenetic group using Pearson’s chi-squared test or Fisher’s exact test for proportions as appropriate.

Functional Category	Virulence Genes	Number (%) of *E. coli* Isolates with Virulence Genes within Phylogenetic Groups							
		A		B1		B2		D	
		Adults	Young Children	Adults	Young Children	Adults	Young Children	Adults	Young Children
		*n* = 68	*n* = 60	*n* = 26	*n* = 10	*n* = 138	*n* = 12	*n* = 64	*n* = 4
**Adhesins**	*fimH*	62 (91.2)	50 (83.3)	26 (100)	10 (100)	134 (97.1)	12 (100)	64 (100)	4 (100)
	*papA*	0	0	4 (15.4)	0	32 (23.2)	6 (50) *	18 (28.1)	4 (100) ***
	*sfaS*	0	0	0	0	4 (2.9)	0	0	0
	*escV*	0	0	1 (0.3)	0	0	0	0	0
**Iron acquisition**	fyuA	32 (47.1) **	12 (20)	20 (76.9)	10 (100)	131 (94.9)	10 (83.3)	42 (65.6)	4 (100)
	*iutA*	30 (44.1) ***	2 (3.3)	14 (53.8)	4 (40)	96 (69.6)	7 (58.3)	44 (68.8)	4 (100)
	*iroN*	4 (5.9)	0	14 (53.8)	2 (20)	88 (63.8) ***	0	4 (6.2)	2 (50) *
	*ireA*	24 (35.3) ***	0	6 (23.1)	4 (40)	40 (29) *	0	17 (26.6)	2 (50)
**Protectins**	*kpsMT* II	22 (32.4) ***	0	14 (53.8)	4 (40)	117 (84.8)	10 (83.3)	48 (75)	2 (50)
	*kpsMT* III	2 (2.9)	0	2 (7.7)	0	12 (8.7)	0	2 (3.1)	0
	*ompT*	8 (11.8) **	0	4 (15.4)	4 (40)	46 (33.3) **	0	4 (6.2)	2 (50) *
	*traT*	34 (50) *	18 (30)	10 (38.5)	4 (40)	98 (71)	11 (91.7)	48 (75)	4 (100)
	*iss*	12 (17.6) ***	0	4 (15.4)	4 (40)	50 (36.2) **	0	4 (6.2)	2 (50) *
**Toxins**	*cnf1*	2 (2.9)	0	10 (38.5) *	0	50 (36.2)	6 (50)	2 (3.1)	0
	*hlyA*	2 (2.9)	4 (6.7)	10 (38.5) *	0	38 (27.5) *	0	0	0
	*east1*	8 (11.8)	21 (35) **	2 (7.7)	0	6 (4.3)	2 (16.7)	12 (18.8)	0
**Biofilm formation**	*agn43*	36 (52.9)	40 (66.7)	20 (76.9) *	4 (40)	124 (89.9)	10 (83.3)	60 (93.8)	4 (100)

*p*-value: *** 0–0.001, ** 0.001–0.01, * 0.01–0.05.

**Table 3:** Prevalence of resistance among the *E. coli* isolates from groups of adults and young children to 17 antimicrobials.

Antimicrobial Agent	Number (%) of *E. coli* Isolates		Test of Independence *p*-Value *
	Adults	Young Children	
	*n* = 296	*n* = 86	
Ampicillin	113 (38.2)	27 (31.4)	0.2506
Amoxicillin/Clavulanic acid	40 (13.5)	3 (3.5)	0.0096 *
Piperacillin	72 (24.3)	5 (5.8)	0.0002 *
Cephalothin	86 (29.1)	24 (27.9)	0.8362
Cefuroxime	20 (6.8)	3 (3.5)	0.3152
Cefotaxime	4 (1.4)	2 (2.7)	0.6206
Streptomycin	137 (46.3)	19 (22.1)	<0.0001 *
Gentamicin	32 (10.8)	9 (10.5)	0.9273
Amikacin	30 (10.1)	7 (8.1)	0.5818
Tetracycline	63 (21.3)	17 (19.8)	0.7610
Doxycycline	46 (15.5)	11 (12.8)	0.5287
Trimethoprim/sulfamethoxazole	33 (11.1)	12 (14)	0.4776
Chloramphenicol	18 (6.1)	0	0.0171
Nalidixic acid	39 (13.2)	8 (9.3)	0.3358
Norfloxacin	8 (2.7)	2 (2.3)	1
Ciprofloxacin	8 (2.7)	2 (2.3)	1
Nitrofurantoin	12 (4.1)	3 (3.5)	1
**Antimicrobial susceptibility characteristic**			
R	224 (75.7)	47 (54.7)	0.0002 *
MDR	90 (30.4)	12 (14)	0.0024 *

R—resistant to at least one agent; MDR—multidrug-resistant. * Statistically significant.

## Supplementary Materials

The following are available online at http://www.mdpi.com/1660-4601/15/4/617/s1, Figure S1: Age distribution among adults. Table S1: List of virulence genes tested in this study. Table S2: Prevalence of the resistance genes among the antimicrobial-resistant *E. coli* isolates from healthy adults and young children.
