# Supplementary material for: Comparison of Commensal Escherichia coli Isolates from Adults and Young Children in Lubuskie Province, Poland: Virulence Potential, Phylogeny and Antimicrobial Resistance
Source: Int J Environ Res Public Health. 2018 Mar 28;15(4):617. doi: 10.3390/ijerph15040617 (PMC5923659; doi:10.3390/ijerph15040617)
Supplement: Supplementary file 1 [file ijerph-15-00617-s001.pdf]

International Journal of Environmental Research and Public Health

Table S1. List of virulence genes tested in this study.

Functional Category	Virulence Gene	Gene Function	*E. coli* Pathotype	References to Papers which Report the PCR Primers and Conditions
**Adhesins**	*fimH*	type 1 fimbriae	ExPEC	16
	papA	P fimbriae	ExPEC	16
	sfaS	S fimbriae	ExPEC	16
	escV	a conservative component of the type III secretion apparatus	EPEC, EHEC	30
	bfpB	bundle forming pilus	EPEC	30
**Iron acquisition**	*fyuA*	yersiniabactin siderophore receptor	ExPEC	16
	iutA	aerobactin siderophore receptor	ExPEC	16
	iroN	salmochelin siderophore receptor	ExPEC	16
	ireA	iron-regulated element, siderophore receptor	ExPEC	16
**Protectins**	*kpsMT* II:	group II capsule	ExPEC	16
	-K1	group II capsule K1 variant	ExPEC	16
	-K2	group II capsule K2 variant	ExPEC	32
	-K5	group II capsule K5 variant	ExPEC	16
	kpsMT III	group III capsule	ExPEC	16
	ompT	outer membrane protein, protease	ExPEC	33
	traT	serum resistance-associated outer membrane protein	ExPEC	16
	iss	increased serum survival	ExPEC	16
**Toxins**	*cnf1*	cytotoxic necrotizing factor 1	ExPEC, NTEC	34
	hlyA	alpha hemolysin	ExPEC	34
	east1	heat stable enterotoxin 1	EAEC	35
	ehxA	enterohemolysin	EPEC, EHEC	35
	stx1	Shiga toxin 1	EHEC	30
	stx2	Shiga toxin 2	EHEC	30
	eltA	heat labile toxin	ETEC	35
	estI	heat stable toxin a	ETEC	35
	estII	heat stable toxin b	ETEC	35
**Biofilm formation**	*agn43*:	antigen 43	ExPEC	36
	-a	antigen 43 allele a	ExPEC	36
	-b	antigen 43 allele b	ExPEC	36
	-K12	antigen 43 allele K12	ExPEC	36

Table S2. Prevalence of the resistance genes among the antimicrobial-resistant *E. coli* isolates from healthy adults and young children.

Antimicrobial Agent	Resistance Gene	Number (%) of Isolates with Resistance Genes		Test of Independence
		Adults	Young Children	*p*-Value
Ampicillin		n = 113	n = 27	
	bla_{TEM}	54 (47.8)	13 (48.1)	0.97312
	bla_{SHV}	6 (5.3)	3 (11.1)	0.3749
Cefotaxim		n = 4	n = 2	
	$bla_{CTX\text{-}M}$	4 (100)	2 (100)	1
Streptomycin		n = 137	n = 19	
	strA/strB	8 (5.8)	3 (15.8)	0.1343
	aadA1	12 (8.8)	4 (21.1)	0.1099
Tetracycline		n = 63	n = 17	
	tetA	15 (23.8)	4 (23.5)	1
	tetB	32 (50.8)	7 (41.2)	0.6668
	tetC	4 (6.3)	0	0.5732

		n = 33	n = 12	
Trimethoprim/ Sulfamethoxazol	*dfrA1*	8 (24.2)	1 (8.3)	0.4069
	dfrA7	18 (54.5)	4 (33.3)	0.8494
	sul1	5 (15.2)	2 (16.7)	1
	sul2	20 (60.6)	3 (25)	0.0758
	sul3	0	0	-
		n = 39	n = 8	
Nalidixic acid	*qnrA*	0	0	-
	qnrB	2 (5.1)	1 (12.5)	0.4364
	qnrS	7 (17.9)	2 (25)	0.6388

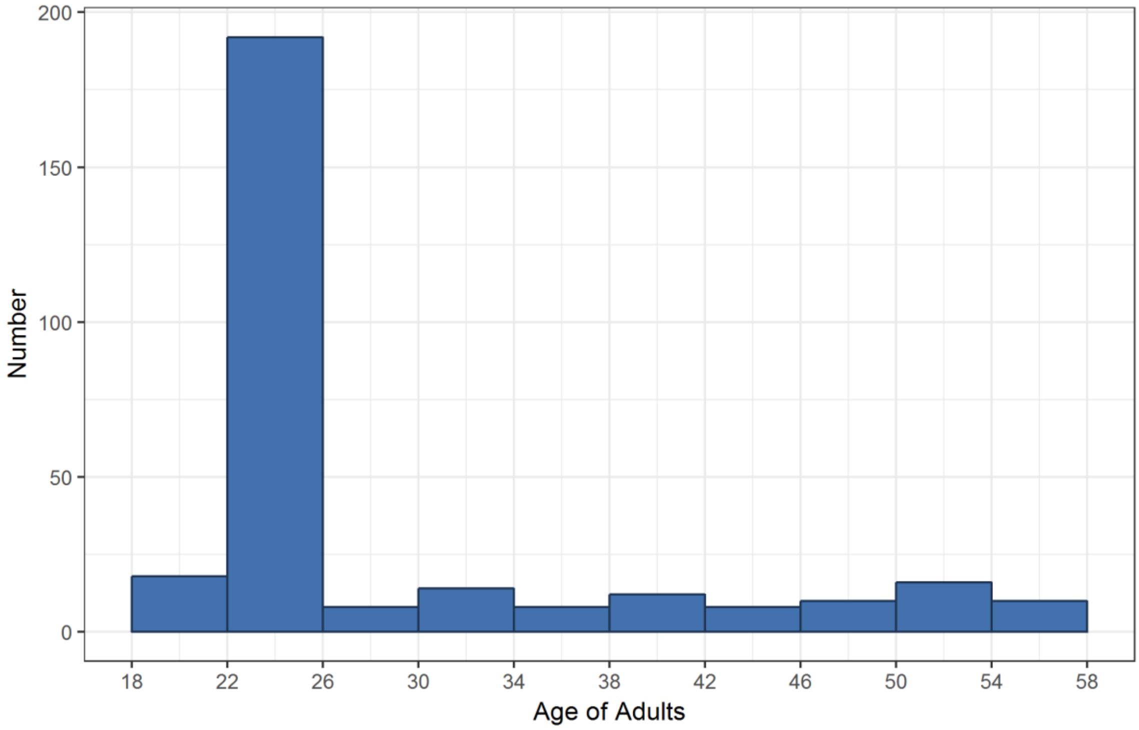

Figure S1. Age distribution among adults.
